# Fusion of a highly N-glycosylated polypeptide increases the expression of ER-localized proteins in plants

**DOI:** 10.1038/s41598-018-22860-2

**Published:** 2018-03-15

**Authors:** Hyangju Kang, Youngmin Park, Yongjik Lee, Yun-Joo Yoo, Inhwan Hwang

**Affiliations:** Division of Molecular and Life Sciences and Division of Integrative Biosciences and Biotechnology, Pohang University of Science and Technology, Pohang, 37673 Korea

## Abstract

Plants represent promising systems for producing various recombinant proteins. One key area of focus for improving this technology is developing methods for producing recombinant proteins at high levels. Many methods have been developed to increase the transcript levels of recombinant genes. However, methods for increasing protein production involving steps downstream of transcription, including translation, have not been fully explored. Here, we investigated the effects of N-glycosylation on protein production and provide evidence that N-glycosylation greatly increases the expression levels of ER-targeted recombinant proteins. Fusion of the extracellular domain (M domain) of protein tyrosine phosphatase receptor type C (CD45), which contains four putative N-glycosylation sites to a model protein, leptin at the C-terminus, increased recombinant protein levels by 6.1 fold. This increase was specific to ER-targeted proteins and was dependent on N-glycosylation. Moreover, expression levels of leptin, leukemia inhibitory factor and GFP were also greatly increased by fusion of M domain at either the N or C-terminus. Furthermore, the increase in protein levels resulted from enhanced translation, but not transcription. Based on these results, we propose that fusing a small domain containing N-glycosylation sites to target proteins is a powerful technique for increasing the expression levels of recombinant proteins in plants.

## Introduction

Recombinant proteins are widely used for various purposes, including the treatment of human diseases^1,2^. Many important studies have led to the current state of recombinant protein production^3,4^. One limitation hampering the production of high-molecular-weight recombinant proteins is that only living organisms can successfully produce recombinant proteins on a commercial scale. Various types of cells have been successfully employed to produce recombinant proteins for commercial purposes. Bacteria were the first organisms to be developed as hosts for recombinant protein production, followed by animal cells and fungi. A recent addition to this list is plant cells, with the promise that recombinant proteins can be produced at low cost and high quality in terms of product safety^5,6^. Indeed, a plant-made pharmaceutical has recently come on the market, and many such products are currently undergoing clinical trials^6^. The overall process for producing recombinant proteins in plants is well established^6–8^.

Many techniques have been developed to produce recombinant proteins in plants. Recombinant genes can be stably inserted into the host nucleus or chloroplast genomes or transiently introduced into plants via *Agrobacterium*-mediated transformation^9,10^. In addition, recombinant proteins can be produced in whole plants or in transgenic cell cultures^11^. One advantage of producing recombinant proteins in plants is the low cost of these systems^6^. Indeed, the facilities required for plant growth can be less costly than those for animal cell culture^12^. However, the overall cost of producing recombinant proteins depends on many factors. One such factor is the expression levels of proteins in plant cells. Many expression vectors have been designed to produce proteins at high levels^13^. Most of these approaches largely rely on the presence of high transcript levels. One such technique is based on the use of plant RNA viruses^14^. In this vector system, target gene transcripts are amplified via RNA-based RNA polymerization. Another high-level production system involves chloroplast transformation, in which recombinant genes are inserted into the chloroplast genome. Using this approach, recombinant proteins can comprise up to 70% of total leaf proteins^15^. Another component incorporated into high-level expression vectors is a transcription terminator, which helps stabilize the transcripts of interest^16^. Obtaining high transcript levels is crucial for high-level protein production. However, high transcript levels are not necessarily associated with high levels of protein production. The nucleotide sequence of the 5′ untranslated region (UTR) upstream of the coding region greatly affects the translational efficiency^17^. Thus, certain expression vectors contain translational enhancers, such as the omega sequence derived from tobacco mosaic virus^18^, or HT 5′ leader from mutated cowpea mosaic virus RNA-2^19^. These enhancers help increase protein levels by enhancing the rate of translation.

The steps downstream of transcript accumulation leading to high levels of recombinant protein production in plants largely remain elusive. These steps involve efficient translation, trafficking, and targeting of the protein to organelles, as well as correct folding. N-glycosylation is a well-known protein modification that occurs in the endoplasmic reticulum (ER). The Asn residue of Asn-Xaa-Ser/Thr (Xaa, any amino acid except Pro) of ER-localized proteins can be glycosylated in the ER. The oligosaccharides on proteins are processed by many enzymes localized to the ER and Golgi apparatus, while these proteins are transported through the Golgi apparatus^20^. N-glycosylation plays a crucial role in protein folding^20,21^. When a nascent polypeptide is folded properly, it can be transported through the secretory pathway. By contrast, misfolded proteins in the ER are degraded by the 26S proteasome in the cytosol via ER-associated degradation (ERAD)^22^. Thus, N-glycosylation (involved in protein folding in the ER) may affect the expression levels of proteins. Consistent with this idea, cases have been reported that the deletion of N-glycosylation sites significantly reduces the expression levels of glycoproteins^23,24^. Conversely, in yeast, cutinase protein levels increased 5-fold and 1.8-fold when an N-glycosylation site was added to the N- or C-terminal region, respectively^25^. However, introducing N-glycosylation sites to recombinant proteins does not always lead to higher levels of protein production. In the case of the propeptide of recombinant *Pseudomonas aeruginosa* elastase (rPAE), N-glycosylation at amino acid position 51 or 93 increased protein levels, whereas N-glycosylation at amino acid position 11 or 127 reduced protein levels^26^. Thus, the effect of N-glycosylation on recombinant protein production has not been fully elucidated.

In this study, we investigated the effects of the fusion of a highly N-glycosylated peptide on recombinant protein expression in plants. We fused leptin which is important hormone for regulating obesity as a model protein with a small extracellular domain, the M domain, from CD45, which contains four putative N-glycosylation sites. We provide evidence that fusing the M domain to the C- or N-termini of target proteins greatly increases recombinant protein production in plants. We also demonstrate that the M domain-mediated increase in protein levels (due to increased N-glycosylation) is specific to ER-targeted proteins and is due to increased rates of translation rather than transcription.

## Results

### Fusion of the M domain (containing multiple N-glycosylation sites) increases recombinant protein levels

**Figure 1:**
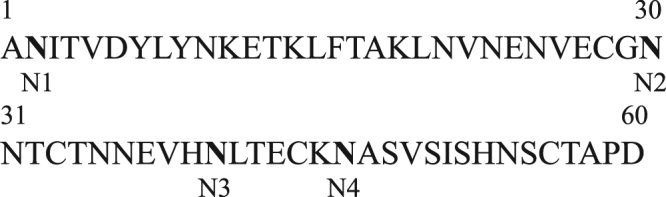
Amino acid sequence of the M domain. The amino acid sequence of human CD45 from Ala231 to Asp290 is shown. The putative N-glycosylation sites of the four Asn residues are indicated by N1 to N4.

**Figure 2:**
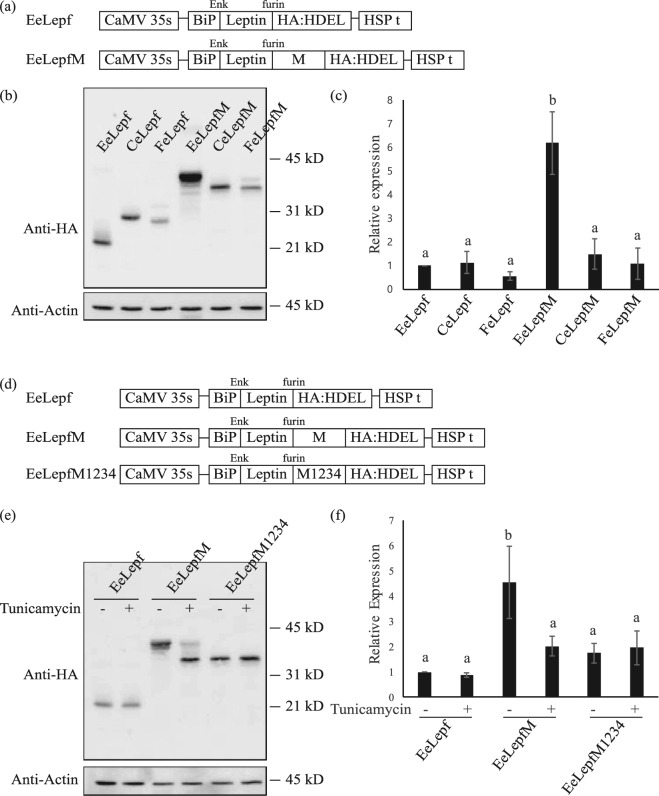
Fusion of the M domain increases the expression levels of recombinant protein. (**a**) Schematic representation of the constructs. All target sequences were expressed under the control of the cauliflower mosaic virus (CaMV) 35 S promoter and heat shock protein (HSP) terminator. ER-targeted proteins contain the leader sequence of BiP. Recombinant proteins targeted to the chloroplast or mitochondria contain the transit peptide of Cab or the presequence of the F1-ATPase gamma subunit, respectively. Enk, enterokinase. (**b**) Western blot analysis of various recombinant proteins. Protein extracts from protoplasts transformed with the indicated constructs were analyzed by western blotting using anti-HA antibody. Actin (detected using anti-actin antibody) was used as a loading control. EeLepf and EeLepfM are ER-targeted proteins, CeLepf and CeLepfM are chloroplast-targeted proteins, and FeLepf and FeLepfM are mitochondria-targeted proteins. (**c**) Quantification of protein levels. The intensity of protein bands from Fig. 2(b) was quantified using the software provided with the LAS4000 image analyzer; values relative to that of ER-targeted EeLepf are shown. Error bars, standard deviation (n = 3). Means with different letters indicate significant difference (Tukey’s test P < 0.05). (**d**) Schematic representation of unglycosylated mutant protein M1234. Four Asn residues were substituted with Gln residues as indicated. (**e**) Analysis of N-glycosylation. Protoplasts transformed with the indicated constructs were incubated for 24 h with or without tunicamycin (10 μg/mL). Protein extracts from transformed protoplasts were analyzed by western blotting using anti-HA antibody. (**f**) Quantification of protein levels. To quantify protein levels, the signal intensity of each band in Fig. 2(e) was measured using the software provided with the LAS4000 system; values relative to that of tunicamycin-untreated EeLepf (EeLepf -) are shown. Three independent experiments were performed. Error bar, standard deviation (n = 3). Means with different letters indicate significant difference (Tukey’s test P < 0.05).

To investigate the effects of N-glycosylation on the expression of recombinant proteins in plants, we selected a 60 amino-acid fragment from positions Ala231 to Asp290 of CD45. This fragment contains four putative N-glycosylation sites, which we named the M domain (Fig. 1). CD45 is a receptor-type tyrosine-protein phosphatase C involved in immune cell activation and differentiation that is heavily glycosylated^27^. For the target protein, we selected leptin, a hormone involved in weight control. The M domain and the small epitope HA (for immunodetection) were fused to the C-terminus of leptin (Fig. 2a). The leptin-M-HA fusion construct (*eLepfM*) was subsequently fused to the BiP leader sequence^28^, Cab transit peptide^29^, or presequence of the F1-ATPase gamma subunit^30^ to induce targeting to the ER, chloroplast, and mitochondria, respectively. Signal peptide, transit peptide, and presequence used in this study are 34, 87, and 77 amino acids long, respectively. After proper targeting into their target organelles, these signal sequences are cleaved, and 7, 35, and 29 amino acids of signal peptide, transit peptide and presequence, respectively, remain in leptin-HA or leptin-M-HA fusion proteins. For the ER-targeted construct, an ER retention signal, HDEL, was additionally fused to the C-terminus of the construct. As controls, we generated constructs without the M domain for the three organelle-targeted constructs. We transformed these six constructs into Arabidopsis protoplasts and measured the expression levels of the recombinant proteins at 24 h after transformation. We separated protein extracts from the protoplasts by SDS-PAGE and analyzed them by western blotting using anti-HA antibody. The apparent molecular weights of all proteins used in this study were larger than the expected molecular weights. Acidic amino acid residues could reduce protein mobility in SDS-PAGE^31^. Indeed, all constructs contain the enterokinase cleavage site, DDDDK.

ER- and chloroplast-targeted fusion proteins without the M domain were expressed at comparable levels, whereas the expression level of the mitochondria-targeted fusion protein was half that of the ER- and chloroplast-targeted fusion proteins. Moreover, their apparent molecular weights indicated that these proteins were successfully targeted to their corresponding target organelles. By contrast, the levels of M domain-containing proteins differed greatly from the levels of these proteins. ER-targeted M domain-containing proteins exhibited as multiple bands due to difference in N-glycosylation. To estimate the expression levels of fusion proteins, intensities of all protein bands except the bottom band were added because the bottom band was considered as non-glycosylated protein based on the protein size. Fusion of the M domain to ER-targeted leptin (EeLepfM) increased protein levels by 6.1-fold compared with the ER-targeted leptin without the M domain (EeLepf). Moreover, the expression level of ER-targeted recombinant protein EeLepfM was 4- and 6-fold higher than that of chloroplast-targeted CeLepfM and mitochondria-targeted FeLepfM, respectively (Fig. 2b,c). In addition, the molecular weight of ER-targeted EeLepfM was significantly higher than that of the mitochondria- and chloroplast-targeted proteins, indicating that ER-targeted EeLepfM was N-glycosylated. As a loading control, we investigated endogenous actin levels using anti-actin antibody and found that the actin levels were identical in all samples.

To investigate the effects of N-glycosylation on protein expression in detail, we generated a construct to express mutant proteins in which Asn residues were substituted with Gln residues. In the ER-targeted leptin-M construct, all four Asn residues in the M domain were substituted with Gln residues to give *EeLepfM1234*. The three ER-targeted constructs (*EeLepf*, *EeLepfM*, and *EeLepfM1234*) were transformed into Arabidopsis protoplasts and incubated in the presence of tunicamycin, an inhibitor of N-glycosylation^32^. The apparent molecular weight of EeLepf and EeLepfM1234 was not altered in the presence of tunicamycin, indicating that these fusion proteins were not N-glycosylated. In addition, tunicamycin treatment did not affect the expression levels of these two fusion proteins, indicating that tunicamycin itself does not affect translation levels. EeLepfM protein levels were greatly reduced by tunicamycin treatment, indicating that N-glycosylation strongly affects protein levels. EeLepfM in the tunicamycin-treated sample migrated significantly more rapidly (in SDS-PAGE) than in the untreated sample, confirming that EeLepfM was heavily N-glycosylated (Fig. 2e,f). These results suggest that N-glycosylation of the M domain increases the levels of M domain-containing fusion proteins.

**Figure 3:**
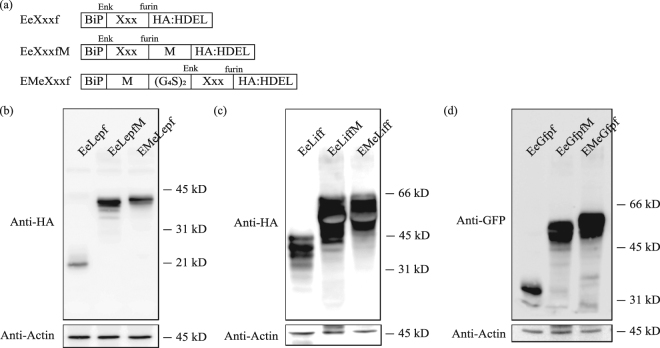
M domain fusion generally increases protein expression levels regardless of the position. (**a**) Schematic representation of constructs with or without the M domain. (G_4_S)_2_ indicates a peptide linker containing two copies of a sequence comprising four Gly and one Ser residue. Xxx is model protein including as leptin (Lep), LIF (Lif), GFP (Gfp). (**b–d**) Expression levels of fusion proteins. Protein extracts from transformed protoplasts with the indicated constructs were analyzed by western blotting using anti-HA (**b**,**c**) or anti-GFP antibodies (**d**). Actin was used as a loading control.

To investigate the effects of the M domain on the expression levels of proteins in general, we used human leukemia inhibitory factor (LIF) and green fluorescent protein (GFP) as target proteins. At the same time, to investigate whether the M domain-induced increase in protein levels has a positional effect, we fused the M domain to the N-terminus of the target protein leptin, LIF and GFP. As controls, we generated constructs, which produces target proteins without the M domain (Fig. 3a). We transformed these 9 constructs into Arabidopsis protoplasts and subjected protein extracts from the protoplasts to SDS-PAGE and western blot analysis using anti-HA antibody. Expression levels of M domain-containing proteins were higher than those of each control protein. LIF is highly glycosylated protein and exhibits a wide range of variation in molecular weight from 20 kD to over 37 kD depending on the host cells^33^. In the cases of leptin and LIF, C-terminal M domain fusion gave higher expression than N-terminal M domain fusion. These results indicate that the M domain increases the levels of recombinant proteins regardless of position, although the degree of increase was dependent on the target proteins (Fig. 3b–d).

### N-glycosylation of the M domain increases recombinant protein levels, which is strongly dependent on the presence of individual N-glycosylation sites and their combination

**Figure 4:**
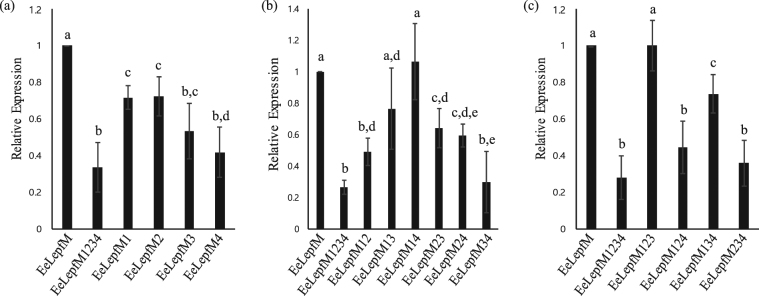
N-glycosylation-mediated increase in fusion protein levels is dependent on individual N-glycosylation sites in the M domain and their combination. (**a**–**c**) Expression levels of various Asn-to-Gln substitution mutants. The indicated Asn-to-Gln mutants were expressed in protoplasts. Protein extracts from transformed protoplasts were analyzed by western blotting using anti-HA antibody. Western blot images are shown in Fig. S1. The signal intensity of protein bands shown in Fig. S1 was quantified using software provided with the LAS4000 image analyzer; relative values to that of the ER-targeted wild-type protein, EeLepfM, are shown. (**a**) Relative levels of single Asn-to-Gln mutants; (**b**) double Asn-to-Gln mutants; (**c**) triple Asn-to-Gln mutants. Error bars, SD (n = 4). Numbers in these mutant constructs indicate Asn residues at the first to fourth position substituted with Gln. Means with different letters indicate significant difference (Tukey’s test P < 0.05).

To obtain insight into the mechanism by which N-glycosylation increases protein levels, we examined whether the N-glycosylation-mediated increase in protein levels is dependent on the number or specific positions of N-glycosylation sites within the M domain. We generated mutant proteins in which one, two, or three Asn residues out of the four N-glycosylation sites were substituted with the corresponding number of Gln residues. For mutant proteins with two or three Asn-to-Gln substitutions, all possible combinations of Asn-to-Gln substitution mutants were generated. We transformed these constructs into protoplasts and examined the expression levels of the mutant proteins by western blot analysis using anti-HA antibody (Fig. S1a–c). The expression levels are shown relative to the expression level of the wild-type M domain-containing protein, EeLepfM (Fig. 4). The protein bands for all four triple mutants were doublet and the bottom band was same size with unglycosylated EeLepfM1234, indicating that all four sites are glycosylated but glycosylation efficiencies differ depending on the potential glycosylation sites (Fig. S1c). The level of EeLepfM1234, in which all four N-glycosylation sites were substituted with Gln residues, was approximately 33% that of the wild-type M domain-containing protein, EeLepfM, indicating that N-glycosylation increased protein levels 3-fold compared with the wild type. The triple mutants containing single N-glycosylation site showed that N-glycosylation at the fourth or second N-glycosylation site (EeLepfM123 and EeLepfM134) increased expression levels whereas N-glycosylation at the third or first N-glycosylation site (EeLepfM124 and EeLepfM234) had no effect (Fig. 4c). Indeed, the expression level of EeLepfM13 which contained the second and fourth N-glycosylation sites was almost two fold higher than that of EeLepfM1234 and had no significant difference from that of fully glycosylated EeLepfM. However, the level of EeLepfM24 which contained the first and third N-glycosylation sites was also significantly increased compared to unglycosylated EeLepfM1234 (Fig. 4b). Moreover, expression levels of all single mutants significantly reduced compared to fully glycosylated EeLepfM, indicating that all N-glycosylation sites had a certain degree of effect on the protein expression (Fig. 4a). These results suggest that any single N-glycosylation site can increase the expression level of proteins and that this increase may depend on the combination or specific location of N-glycosylation sites in the M domain. To confirm that the differences in the levels of these proteins were not due to differences in the transcript levels of the constructs, we performed quantitative RT-PCR analysis and found that the transcript levels of all 16 constructs did not show any statistical difference (Fig. S2).

### N-glycosylated-induced increases in EeLepfM expression levels are due to high rates of translation

To gain insight into the mechanism underlying how N-glycosylation increases protein levels, we examined whether chaperones in the ER contribute to this increase. N-glycosylation is crucial for the proper folding of proteins in the ER, thereby contributing to protein expression^34^. Among these ER chaperones, binding protein (BiP) plays a critical role in N-glycosylation and protein folding^34,35^. To investigate the possible involvement of BiP in the N-glycosylation-mediated increase in protein levels, we examined the endogenous BiP levels of the samples by western blot analysis using anti-BiP antibody. We analyzed protein extracts from protoplasts transformed with glycosylated or unglycosylated constructs by western blotting using anti-BiP antibody. BiP levels did not differ between the two samples, indicating that BiP does not play a direct role in the N-glycosylated-induced increase in protein levels (Fig. S1d).

**Figure 5:**
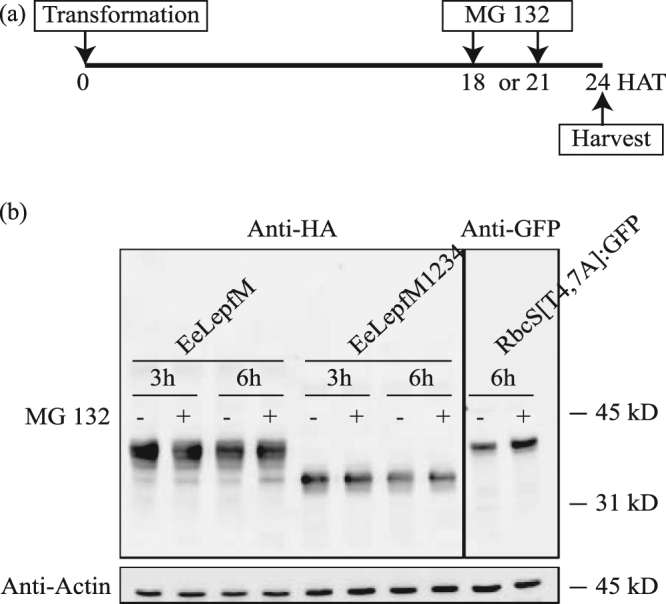
Low expression levels of unglycosylated proteins are not caused by ER-associated degradation. (**a**) Scheme of experimental design. MG132 was added to the protoplast incubation medium at 18 or 21 h after transformation of the indicated constructs, and the protoplasts were further incubated for 6 or 3 h, respectively. HAT, h after transformation. (**b**) Western blot analysis of proteins. Protein extracts were prepared from protoplasts harvested at 24 h after transformation and analyzed by western blotting using anti-HA antibody. RbcS[T4,7 A]:GFP was analyzed using anti-GFP antibody as a positive control for 26S proteasome-mediated degradation and MG132 treatment. Actin (detected using anti-actin antibody) was used as a loading control.

N-glycosylation plays a critical role in protein folding in the ER. Thus, unglycosylated proteins may not be efficiently folded in the ER. The unfolded proteins are removed from the ER and degraded in the cytosol via a mechanism known as ERAD involving ubiquitination and the 26S proteasome^35^. We therefore investigated whether unglycosylated fusion proteins are subject to ERAD. Protoplasts transformed with N-glycosylated *EeLepfM* or unglycosylated *EeLepfM1234* were treated with MG132, an inhibitor of 26S proteasome-mediated degradation, at 18 or 21 h after transformation and further incubated for 6 or 3 h, respectively. As a positive control for MG132 treatment, protoplasts transformed with *RbcS[T4*,*7 A]:GFP*, a GFP fusion construct expressing a mutant form of the RbcS transit peptide defective in protein import into chloroplasts, were treated with MG132 at 18 h after transformation and further incubated for 6 h^36^. RbcS[T4,7 A]:GFP is ubiquitinated and degraded by the 26S proteasome in the cytosol. We analyzed protein extracts from the transformed protoplasts by western blotting using anti-HA antibody; RbcS[T4,7 A]:GFP was analyzed with anti-GFP antibody as a control. RbcS[T4,7 A]:GFP levels were higher in the presence versus the absence of MG132, indicating that, under our conditions, MG132 inhibits 26S proteasome-dependent proteolysis. However, EeLepfM and EeLepfM1234 protein levels did not differ regardless of MG132 treatment (Fig. 5), indicating that ERAD does not play a role in the differences in protein levels of unglycosylated EeLepfM1234 versus N-glycosylated EeLepfM.

**Figure 6:**
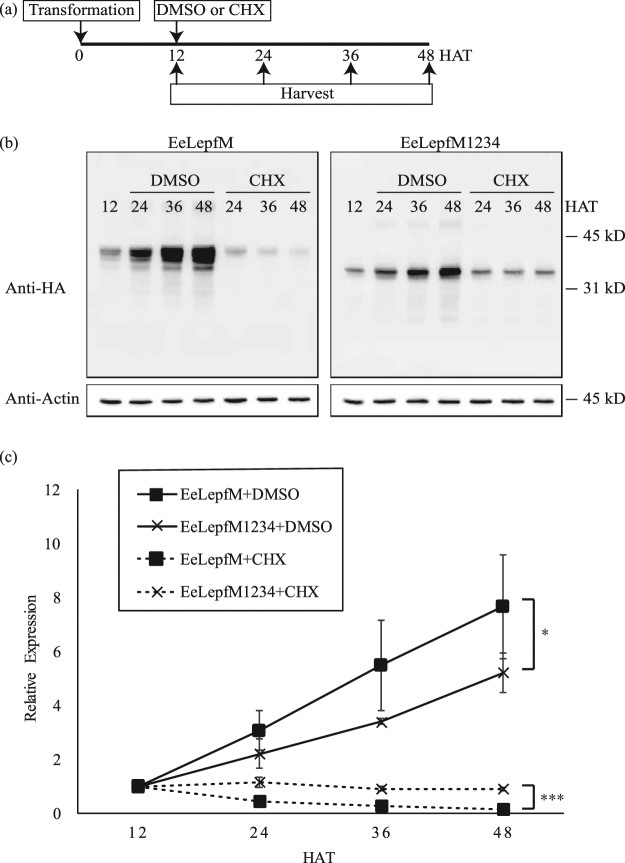
The translation rate of the M domain-containing recombinant protein is higher than that of the mutant fusion protein containing an unglycosylated M domain. (**a**) Scheme of experimental design. Cycloheximide (CHX) or DMSO (as a control) was added to the protoplast incubation medium at 12 h after transformation of the indicated constructs, and protoplasts were harvested at the indicated time points. (**b**) Western blot analysis of protein levels. Protein extracts from protoplasts were analyzed by western blotting using anti-HA antibody. Actin (detected using anti-actin antibody) was used as a loading control. (**c**) Quantification of protein levels. The signal intensities of the protein bands in Fig. 6(b) were measured using software provided with the LAS4000 image analyzer and are expressed as relative values to that of the corresponding constructs in 12 HAT samples Error bars, SD (n = 3). Repeated-measures two-way analysis of variance (ANOVA) detected a significant expression level difference between EeLepfM and EeLepfM1234 in the absence (DMSO) or presence (CHX) of cycloheximide (*p < 0.05; ***p < 0.001).

Next, we examined whether protein stability contributes to the higher levels of EeLepfM than EeLepfM1234 in the ER. We treated protoplasts transformed with *EeLepfM* or *EeLepfM1234* with cycloheximide, an inhibitor of translation^37^, at 12 h after transformation. We prepared protein extracts every 12 h after cycloheximide treatment until 48 h after transformation and analyzed protein levels by western blotting using anti-HA antibody. Unglycosylated EeLepfM1234 was maintained at the comparable level until 48 h after transformation (92%), whereas the protein levels of EeLepfM at 48 h after transformation were reduced to only 16% those at 12 h after transformation (Fig. 6), indicating that EeLepfM1234 is more stable than EeLepfM in the ER. These results suggest that protein stability does not contribute to the difference in protein levels between EeLepfM1234 and EeLepfM.

The results in Figs 5 and 6 raised the possibility that the higher levels of EeLepfM versus EeLepfM1234 resulted from a higher rate of translation. We therefore examined the relative expression of EeLepfM versus EeLepfM1234 at various times after protoplast transformation. The level of EeLepfM protein increased more rapidly than that of EeLepfM1234 (Fig. 6). To get direct evidence that N-glycosylation enhances translation rate, *in vitro* translation was performed using transcription-translation coupled wheat germ extracts system. Proteins produced in wheat germ extracts was not translocated into microsomal membranes because of the absence of signal recognition particle (SRP). Protein levels produced in wheat germ extracts were similar regardless of N-glycosylation site mutations, indicating that translation occurs at the same rate if protein is not N-glycosylated (Fig. S3). These results imply that the process involved in N-glycosylation of proteins at the ER results in the higher levels of protein production.

## Discussion

In this study, we examined the effect of N-glycosylation on recombinant protein levels in plants and provide compelling evidence that N-glycosylation greatly increases the level of recombinant protein production in plants. To investigate the effects of N-glycosylation on recombinant protein levels, we took the approach of fusing a small domain containing multiple N-glycosylation sites to target proteins instead of introducing N-glycosylation sites to the target proteins by site-directed mutagenesis. The advantage of our approach is that the N-glycosylation sites are added in an extra domain, and thus are not expected to affect the structure of the target proteins we used. Elliot and their colleagues introduced multiple combinations of N-glycosylation sites in native leptin sequence^38^. When those leptins were expressed in Chinese hamster ovary (CHO) cells secretion of some, not all, glycosylated leptin was increased and GE-Leptin L4 having 5 N-glycosylation sites exhibited greater and prolonged effect on reducing body weight. However, introducing N-glycosylation sites into native protein did not always result in high expression or bioactivity. In this approach, the N-glycosylation domain is fused to the target proteins as a separate domain via a small linker. One advantage of this approach is that the recombinant proteins are not mutated within the target sequence. However, there is a caveat: the recombinant proteins contain an extra domain. Thus, if the production of only the native target protein is desired, the extra domain should be removed from the recombinant protein after it is produced in plants.

Many approaches have been taken to increase protein levels in plants. Most approaches focus on increasing transcript levels using either strong promoters or viral RNA or DNA amplification systems^39,40^. Increasing transcript levels contribute greatly to increasing protein levels. However, when recombinant proteins are produced in cells, many steps occur downstream of transcription. These steps may also contribute to high levels of protein production. Indeed, we found that the mutations at N-glycosylation sites in the M domain of human CD45 did not alter the transcript levels of recombinant genes when fused to target proteins. This result raised the possibility that the N-glycosylation of the M domain plays a role somewhere downstream of transcription to increase protein levels.

One crucial step in protein production downstream of transcription is translation on ribosomes. Thus, translation efficiency should strongly affect the levels of proteins in the cell. However, higher levels of transcripts do not necessarily lead to the higher levels of proteins^41,42^. Moreover, the 5′ UTR upstream of the coding region greatly affects translational efficiency^17,43^; 5′ UTR sequences that affect translational efficiency include specific sequence motifs such as omega sequences, translational enhancers, and internal ribosome entry sites^18,44,45^. Insertion of the omega sequence leads to a several-fold increase in protein levels^18^. The steps after translation on ribosomes include targeting to organelles (in the case of organellar proteins), folding, maturation, and storage in the cell^46^. All of these steps should affect protein levels. In the current study, the presence of the M domain increased the levels of ER-targeted proteins, but not chloroplast- or mitochondria-targeted proteins. These results strongly suggest that N-glycosylation is involved in the increase in protein levels. Consistent with this idea, substituting four Asn residues with Gln residues in the M domain eliminated the M domain-mediated increase in protein levels. We cannot completely exclude the possibility that low levels of proteins fused with non-glycosylated M domain is due to the alteration of the M domain structure. At least, proteins fused with non-glycosylated M domain was more stable in the ER and not degraded. Fusion of the M domain to either the N- or C-termini of target proteins increased protein levels. Moreover, not all N-glycosylation sites were necessary to increase protein levels. In fact, even the addition of a single N-glycosylation site was sufficient to increase protein levels. This finding suggests that the increase in protein levels is not dependent on the number of N-glycosylation sites. Moreover, M domain- and its mutants-fused leptins except glycosylation-free mutant exhibited as multiple bands in western blot results, indicating that the efficiency of N-glycosylation differs depending on the potential N-glycosylation sites. In fact, the location of the N-glycosylation sites in the M domain or their combination (when multiple N-glycosylation sites were present in the M domain) was critical for increasing protein levels or glycosylation pattern. Thus, it was hard to expect the efficiency of glycosylation at each site. Currently, we were unable to propose any rule about the effects of N-glycosylation on the increase in protein levels. Similarly, N-glycosylation increases protein levels in yeast and *Pseudomonas aeruginosa*^25,26^. However, depending on the position of the N-glycosylation sites, protein levels can also be reduced by N-glycosylation^26^. Thus, the exact effect of N-glycosylation on protein levels should be determined experimentally.

What is the underlying mechanism for the N-glycosylation-mediated increase in protein levels in plants? N-glycosylation of ER proteins is crucial for protein folding^20^. We eliminated the possibility that N-glycosylation contributes to the stability of recombinant proteins in the ER; in fact, N-glycosylated EeLepfM was less stable than unglycosylated EeLepfM1234 in the ER. By contrast, the accumulation rates of N-glycosylated proteins were much higher than those of unglycosylated proteins. One possibility is that N-glycosylation increases the translation rate. *In vitro* translation analysis revealed that whether proteins have potential N-glycosylation sites or not they were expressed at the same levels when they were produced as non-glycosylated forms in wheat germ extracts. These results suggest that the nucleotide or amino acid sequence differences do not affect the translation rate and raise the possibility that a process involved in N-glycosylation at the ER is crucial for high translation rate. However, how N-glycosylation affects the translation rate is currently unclear. Perhaps N-glycosylation facilities the folding of nascent proteins in the ER, which in turn leads to a higher rate of translation on ribosomes. The exact mechanism by which N-glycosylation enhances the translation rate of ER proteins should be further investigated in the future.

High levels of recombinant protein expression are essential for the successful use of plants as protein production systems. Much effort has been devoted to increasing the transcript levels of target genes to increase recombinant protein levels^8^. In this study, we developed a new approach in which N-glycosylation is used to increase recombinant protein levels in plants. Fusion of a domain containing multiple N-glycosylation sites to the N- or C-termini of target proteins greatly increases protein levels.

## Experimental Procedures

### Plant materials and growth conditions

Arabidopsis (*Arabidopsis thaliana* ecotype, Col-0) plants were grown on B5 plates in a growth chamber at 20 °C to 22 °C under a 16 h/8 h light/dark cycle. Leaf tissues from 2-week-old plants were used for protoplast isolation.

### Plasmid construction

The mature peptide region of mouse leptin cDNA (NM_008493.3) was used. The DNA fragment encoding the M domain was synthesized by repetitive polymerase chain reaction (PCR), and all combinations of Asn-to-Gln substitution mutants were generated by PCR-based site-directed mutagenesis. The mature peptide region of human *LIF* cDNA (NM_002309.4) was amplified by PCR. Enterokinase and furin cleavage sites were included in the primer used for leptin amplification. The HA epitope and ER retention signal HDEL were introduced by the primer used for M domain amplification. The sequences of primers used in this study are listed in Table S1. PCR products of the mature region of leptin and the M domain, including Asn-to-Gln substitution mutants, were sequentially ligated into the vector BiP:HA:CBD:HDEL^28^. To generate chloroplast- and mitochondria-targeted constructs, the Cab transit peptide or the F1-ATPase gamma subunit presequence was amplified by PCR and substituted with BiP in the EeLepf and EeLepfM vector^29,30^. All constructs were generated with the same vector, thus 5′-UTRs were same for all constructs (5′-ATTATTACATCAAAACAAAAA-3′)^17^. The sequences of all PCR products were verified by nucleotide sequencing.

### Transient expression, chemical treatments, and western blot analysis

Plasmids were introduced into protoplasts by polyethylene glycol-mediated transformation^47^. Protein extracts were prepared at 24 h after transformation or at the indicated time points^48^. The protoplasts were treated with tunicamycin (10 μg/mL; Sigma-Aldrich, St. Louis, MO) immediately after transformation. Cycloheximide (50 μg/mL; Sigma-Aldrich, St. Louis, MO) treatment was applied at 12 h after transformation. Protein extracts were analyzed by western blotting with anti-HA (Roche Diagnostics, Indianapolis, IN), anti-actin (MP Biomedicals, Solon, OH), anti-GFP (Bio-Application, Pohang, Korea), or anti-BiP antibodies^47–49^. The protein blots were developed with an ECL kit (Amersham Pharmacia Biotech, Piscataway, NJ), and images were obtained using an LAS4000 image analyzer (Fujifilm, Tokyo, Japan). To quantify expression levels, intensity of protein bands in western blot images was measured using Multi-Gauge program equipped to LAS4000 image analyzer (FUJIFILM, Tokyo, Japan). Band intensities were added in cases of glycosylated proteins except the bottom band that was considered as the non-glycosylated form.

### Total RNA isolation and quantitative RT-PCR analysis of transcript levels

Total RNA was extracted from protoplast after PEG transformation using the Ambion Phenol-free total RNA isolation kit and treated with TURBO DNase (Ambion). cDNA was synthesized using the high-capacity cDNA reverse transcription kit (Applied Biosystems). Power SYBR Green PCR Master Mix (Applied Biosystems) was used to detect transcript levels. *ACTIN2* was used as an endogenous control. The PCR mixture (20 μL) contained 50 ng of template, 0.5 mM forward and reverse primers, and 1X SYBR Mix. The PCR conditions were as follows: initial denaturation at 95 °C for 10 min, followed by 40 cycles of 95 °C for 15 s and 60 °C for 1 min. To confirm specific amplification, a melting curve was generated by heating at 95 °C for 15 s and then at 60 °C for 1 min, and then increasing the temperature by 0.3 °C every 15 s up to 95 °C.

### *In vitro* translation assay

For *in vitro* translation, DNA fragments from start codon to stop codon of EeLepfM and EeLepfM1234 were ligated into pCS2++ vector harboring SP6 promoter and SV40 terminator. PCR was performed to generate linearized DNA using primers, SP6 promoter-339bp-F and SV40 terminator-R. *In vitro* translation assay was performed using transcription and translation coupled wheat germ extracts system following manufacture’s protocol (Promega).

### Statistical analysis

Data in graphs are expressed as means and error bars represent standard deviation (SD). To quantify protein expression levels, we measured intensity of protein bands in western blot images from at least three biological replicates. Statistical analyses were performed using one-way analysis of variance (ANOVA) followed by a post hoc Tukey’s test with P < 0.05 to be considered significant or repeated-measures two-way ANOVA.

### Data availability

All data generated or analyzed during this study are included in this published article (and its Supplementary information files).

## Electronic supplementary material

supplementary information
