# Supplementary material for: Fusion of a highly N-glycosylated polypeptide increases the expression of ER-localized proteins in plants
Source: Sci Rep. 2018 Mar 15;8:4612. doi: 10.1038/s41598-018-22860-2 (PMC5854594; doi:10.1038/s41598-018-22860-2)
Supplement: Supplementary file 1 — supplementary information [file 41598_2018_22860_MOESM1_ESM.docx]

**Supplementary information**

**Fusion of a highly N-glycosylated polypeptide increases the expression of ER-localized proteins in plants**

Hyangju Kang, Youngmin Park, Yongjik Lee, Yun-Joo Yoo, Inhwan Hwang*

Division of Molecular and Life Sciences and Division of Integrative Biosciences and Biotechnology, Pohang University of Science and Technology, Pohang, 37673, Korea

*To whom correspondence should be addressed.

Tel: 82-54-279-2128

Email: ihhwang@postech.ac.kr

**Figure S1. BiP levels are not affected by the differences in N-glycosylation patterns of M domain fusion proteins in protoplasts.**

(**a**–**c**) Western blot analysis of single (**a**), double (**b**), and triple (**c**) Asn-to-Gln substitution mutants. Protein extracts from protoplasts transformed with the indicated constructs were analyzed by western blotting using anti-HA antibody. Endogenous BiP was detected using anti-BiP antibody. Actin (detected using anti-actin antibody) was used as a loading control.

(**d**) Quantification of BiP levels. Signal intensities of BiP bands were measured using software provided with the LAS4000 image analyzer and are expressed as relative values to that of the BiP band from protoplasts transformed with the wild-type M domain-containing construct, *EeLepfM*. Error bars, SD (n = 3).

**Figure S2. Transcript levels of various M fusion constructs, as determined by quantitative RT-PCR.**

Total RNA was prepared from protoplasts transformed with the indicated constructs and used for quantitative RT-PCR analysis using *leptin* primer sets. Mean values of relative levels of transcript compared to fully glycosylated *EeLepfM* were presented in the graph. Error bar, SD (n=3 for M to 14; n=2 for 23 to 1234). M indicates the construct fused with the WT M domain while each number indicates constructs fused with the M domain which has mutation at the indicated N-glycosylation sites. One-way ANOVA result showed that the difference in the transcript levels between M domain-fused leptins including mutants was not significant (P > 0.05).

**Figure S3. *In vitro* translation analysis of EeLepfM and EeLepfM1234.**

Western blot analysis of *in vitro* translated proteins in wheat germ extracts. Protein extracts were prepared at the indicated time points from reaction mixtures that had been incubated with the indicated linearized DNA and analyzed by western blotting using anti-HA antibody. Protein extracts were prepared from the reaction mixture with no DNA and used as control.

**Figure S4. Full blot scans.**

Full blot scans from **Figure 5**. Western blotting membrane was firstly detected using anti-HA antibody (**a**). After stripping, same membrane was detected using anti-GFP antibody (**b**).

**Table S1. Primers used in this study**

**Figure S1. BiP levels are not affected by the differences in N-glycosylation patterns of M domain fusion proteins in protoplasts.**


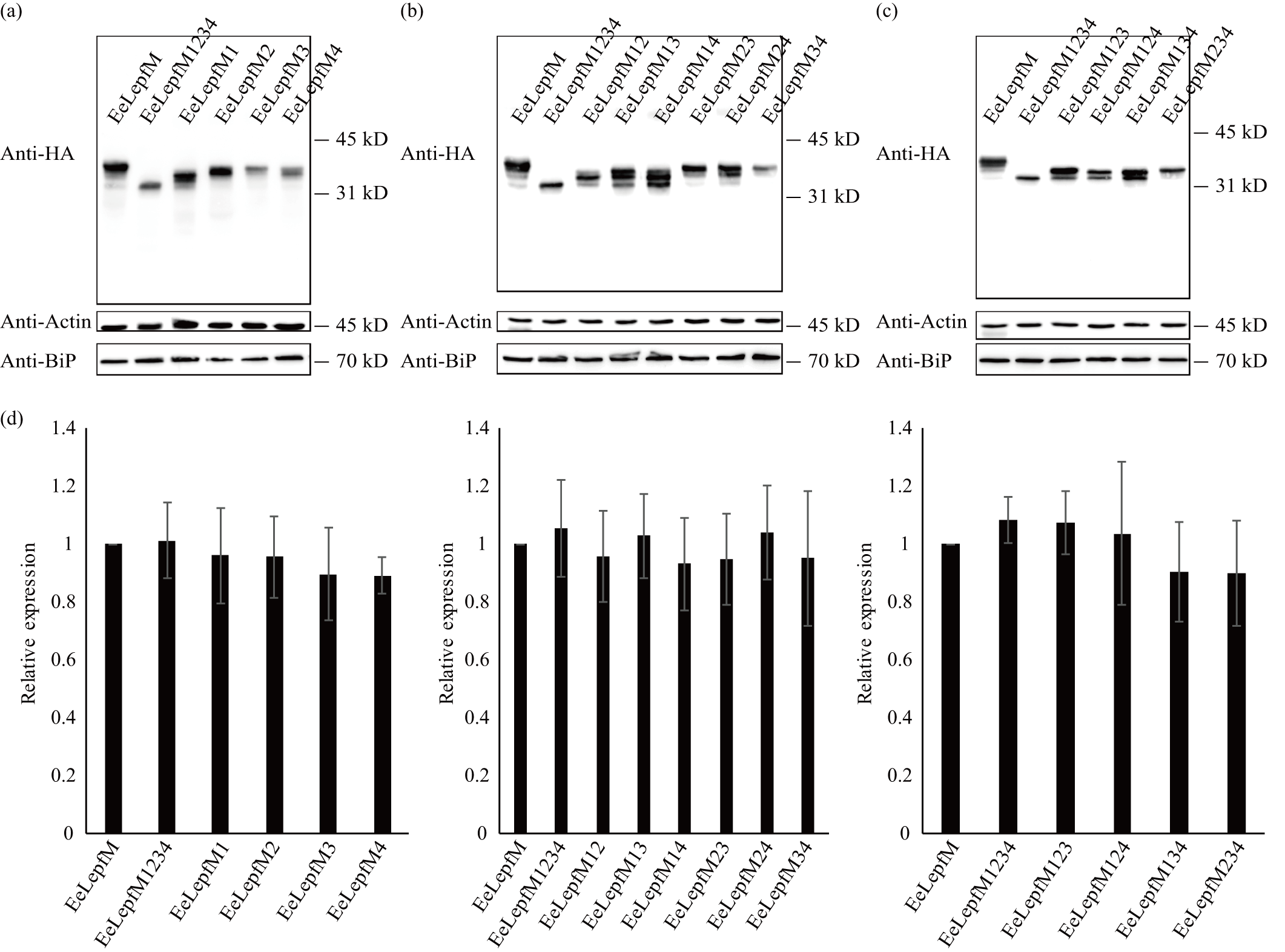


**Figure S2. Transcript levels of various M fusion constructs, as determined by quantitative RT-PCR.**


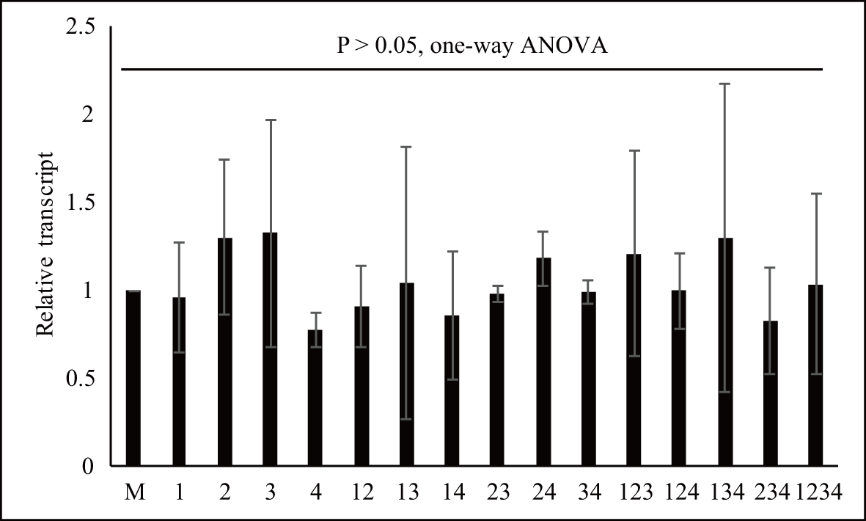


**Figure S3. *In vitro* translation analysis of EeLepfM and EeLepfM1234.**


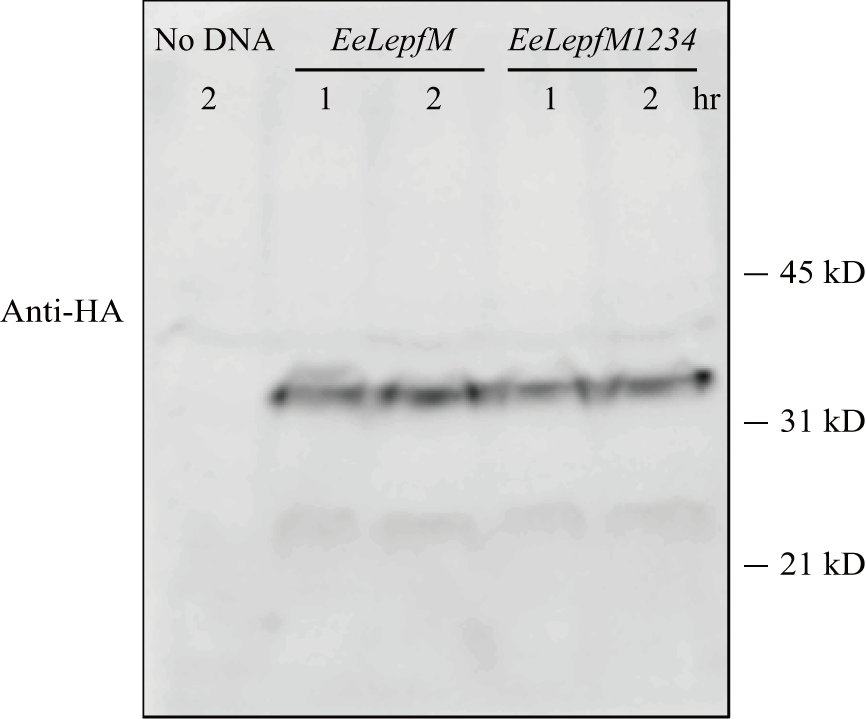


**Figure S4. Full blot scans.**


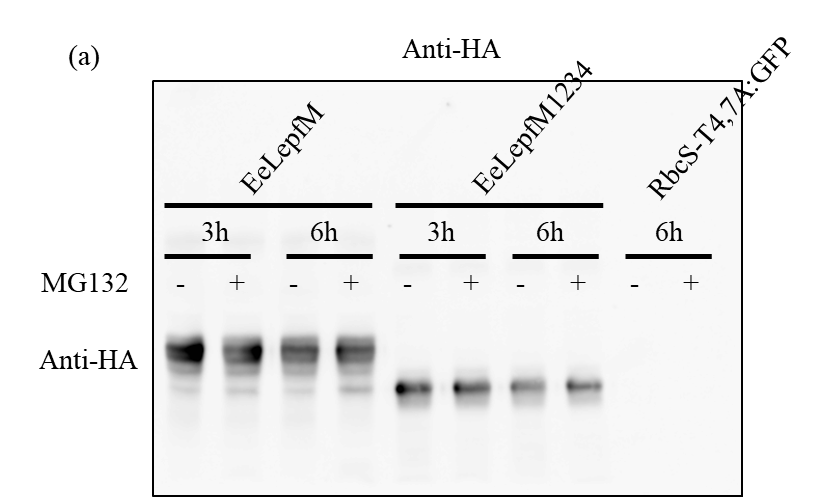


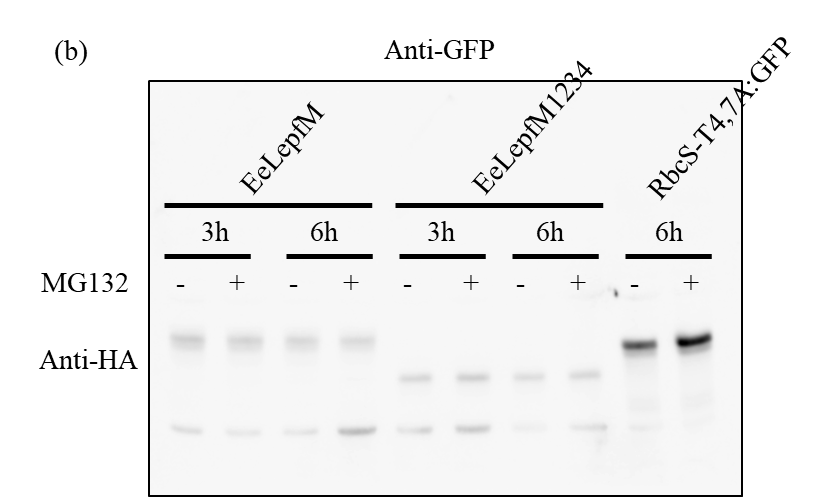


**Table S1. Primers used in this study**

| Name | Sequence (5’ to 3’) |
| --- | --- |
| BamHI-Ek-leptin-F | GGATCCAAGATGATGATGATAAGGTGCCTATCCAGAAAGTCCAGGAT |
| leptin-furin-SpeI-R | ACTAGTTCGCCTGACACGGCATTCAGGGCTAACATCCAACTG |
| hspt-R | GAATTCCTTATCTTTAATCATATT |
| M-3-HA-HDEL-F | TCATAATTCATGTACTGCTCCTGATTACCCATACGATGTTCCAGATTACGCTTCCCACGATGAGCTCTAGCTCGAGATATGAAGATGAAGATGAAATATT |
| M-2-F | AATGTGGAAACAATACTTGCACAAACAATGAGGTGCATAACCTTACAGAATGTAAAAATGCGTCTGTTTCCATATCTCATAATTCATGTACTGCTCCTGA |
| SpeI-M-1-F | ACTAGTGCAAACATCACTGTGGATTACTTATATAACAAGGAAACTAAATTATTTACAGCAAAGCTAAATGTTAATGAGAATGTGGAATGTGGAAACAATACTTGCACAA |
| SpeI-HA-F | ACTAGTTACCCATACGATGTTCCAGATTAC |
| XbaI-Cab-F | TCTAGAATGGCGTCGAACTCGCTTATGAGC |
| Cab-BamHI-R | GGATCCTCTCTGACTCTTTGTA |
| XbaI-F1-F | TCTAGAATGGCAATGGCTGTTTTCCGTCGC |
| F1-BamHI-R | GGATCCTCTGAACTGCTCTAAGCTTGGAAG |
| SpeI-M-F | ACTAGTGCAAACATCACTGTGGAT |
| SpeI-M-N2Q-F | ACTAGTGCACAAATCACTGTGGAT |
| M-N30Q-F | GTGGAATGTGGACAAAATACTTGCACA |
| M-N30Q-R | TGTGCAAGTATTTTGTCCACATTCCAC |
| M-N40Q-F | AATGAGGTGCATCAACTTACAGAATGT |
| M-N40Q-R | ACATTCTGTAAGTTGATGCACCTCATT |
| M-N46Q-F | ACAGAATGTAAACAAGCGTCTGTTTCC |
| M-N46Q-R | GGAAACAGACGCTTGTTTACATTCTGT |
| M-N40,46Q-F | AACAATGAGGTGCATCAACTTACAGAATGTAAACAAGCGTCTGTTTCCATA |
| M-N40,46Q-R | TATGGAAACAGACGCTTGTTTACATTCTGTAAGTTGATGCACCTCATTGTT |
| BamHI-M-F | GGATCCCGATGGCAAACATCACTGTGGATTACTTA |
| M-GS2-SpeI-R | ACTAGTTGATCCACCACCAGACCCACCTCCACCATCAGGAGCAGTACATGAATTAT |
| BamHI-Ek-LIF-F | GGATCCAAGATGATGATGATAAGATGAGCCCCCTCCCCATCACCCCT |
| LIF-RVRR-SpeI-R | ACTAGTTCGCCTGACACGGAAGGCCTGGGCCAACACGGCGAT |
| SpeI-Ek-Lif-F | ACTAGTGATGATGATGATAAGATGAGCCCC |
| Lif-RVRR-HA-R | GTAATCTGGAACATCGTATGGGTATCGCCTGACACGGAAGGCCTGGGC |
| GFP-HDEL-stop-XhoI-R | CTCGAGCTAGAGCTCATCGTGCTTGTACAGCTCGTCCATGCCGAG |
| GFP-fu-SpeI-R | ACTAGTTCGCCTGACACGCTTGTACAGCTCGTCCATGCCGAG |
| SpeI-ek-GFP-F | ACTAGTGATGACGACGATAGGTGAGCAAG |
| SP6 promoter-339bp-F | TGCCCATTCATATCCGTTCT |
| SV40 terminator-R | GTTGTTAACTTGTTTATTGCAGCT |
| AtACT2-5’ | TATGAATTACCCGATGGGCAAG |
| AtACT2-3’ | TGGAACAAGACTTCTGGGCAT |
| leptin- F-qRT1-F | TCGGTATCCGCCAAGCAGTGCCTATCCAGAAAGTCCA |
| leptin- R-qRT1-R | GGTGAAGCCCAGGAATGAAGGCATTCAGGGCTAACATCCA |
